# Ethyl 3-oxo-2-(2-phenyl­hydrazinyl­idene)butano­ate: a re-determination

**DOI:** 10.1107/S1600536811002200

**Published:** 2011-01-22

**Authors:** Satish Chandra Gupta, Deo Kumar Mandal, Asha Rani, Anup Sahay, Satya Murti Prasad

**Affiliations:** a Department of Physics, Ranchi University, Ranchi 834 008, India; b Department of Chemistry, Bihar University, Muzzafarpur 842 002, India

## Abstract

The previous crystallographic studies [Wang *et al.* (2005 ▶). *Huaxue Yanjiu* **16**, 29–32; Wang *et al.* (2007 ▶). *Youji Huaxue*, **27**, 524] of the title compound, C_12_H_14_N_2_O_3_, gave only the unit-cell dimensions and an *R* factor with no other details available: the full structure is presented here. The eth­oxy group is disordered over two orientations with refined occupancies of 0.642 (15):0.358 (15). The nine C atoms and two N atoms of the 1-phenyl-2-(propan-2-yl­idene)hydrazine segment of the mol­ecule are close to being coplanar, with a maximum deviation of 0.0779 (14) Å for the phenyl­amino N atom and an intra­molecular N—H⋯O hydrogen bond generates an *S*(6) ring. In the crystal, pairs of C—H⋯O hydrogen bonds link mol­ecules into inverson dimers, generating *R*_2_^2^(16) loops.

## Related literature

For previous reports of the structure of the title compound, see: Wang *et al.* (2005 ▶, 2007 ▶). For further synthetic details, see: Fernandes *et al.* (1975 ▶). For graph-set analysis of hydrogen bonding, see: Bernstein *et al.* (1995 ▶).
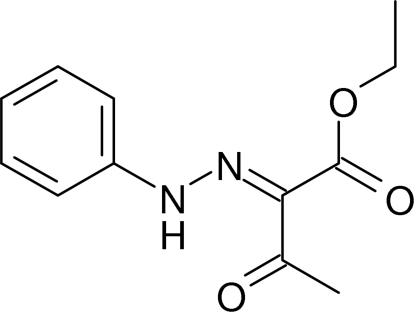


## Experimental

### 

#### Crystal data

C_12_H_14_N_2_O_3_
*M* *_r_* = 234.25
Monoclinic, 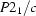

*a* = 8.4375 (9) Å
*b* = 17.551 (2) Å
*c* = 8.242 (1) Å
β = 91.24 (1)°
*V* = 1220.2 (2) Å^3^
*Z* = 4
Cu *K*α radiation
μ = 0.77 mm^−1^
*T* = 293 K
0.2 × 0.16 × 0.12 mm

#### Data collection

Enraf–Nonius CAD-4 diffractometer
2393 measured reflections
2243 independent reflections
1715 reflections with *I* > 2σ(*I*)
*R*_int_ = 0.022
3 standard reflections every 60 min  intensity decay: 0.0%

#### Refinement

*R*[*F* ^2^ > 2σ(*F* ^2^)] = 0.052
*wR*(*F* ^2^) = 0.185
*S* = 1.07
2243 reflections
186 parameters
H-atom parameters constrained
Δρ_max_ = 0.19 e Å^−3^
Δρ_min_ = −0.17 e Å^−3^

### 

Data collection: *CAD-4 EXPRESS* (Enraf–Nonius, 1994 ▶); cell refinement: *CAD-4 EXPRESS*; data reduction: *MolEN* (Fair, 1990 ▶); program(s) used to solve structure: *SHELXS97* (Sheldrick, 2008 ▶); program(s) used to refine structure: *SHELXL97* (Sheldrick, 2008 ▶); molecular graphics: *ORTEP-3* (Farrugia, 1997 ▶); software used to prepare material for publication: *SHELXL97*.

## Supplementary Material

Crystal structure: contains datablocks I, global. DOI: 10.1107/S1600536811002200/hb5790sup1.cif

Structure factors: contains datablocks I. DOI: 10.1107/S1600536811002200/hb5790Isup2.hkl

Additional supplementary materials:  crystallographic information; 3D view; checkCIF report

## Figures and Tables

**Table 1:** Hydrogen-bond geometry (Å, °)

*D*—H⋯*A*	*D*—H	H⋯*A*	*D*⋯*A*	*D*—H⋯*A*
N1—H1⋯O1	0.86	1.92	2.571 (2)	131
C2—H2⋯O1^i^	0.93	2.53	3.430 (3)	163

Symmetry code: (i) 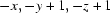.

**Fig. 1.**
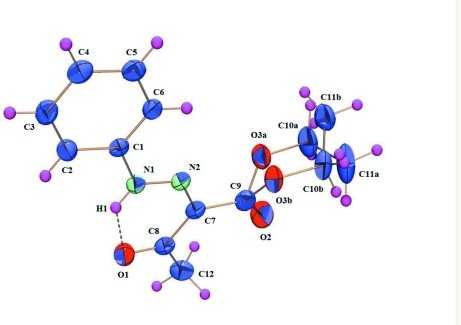
The structre of (I) with 30% probability displacement ellipsoids for non-hyrogen atoms showing the disordered ethoxy group O3—C10—C11.

**Fig. 2.**
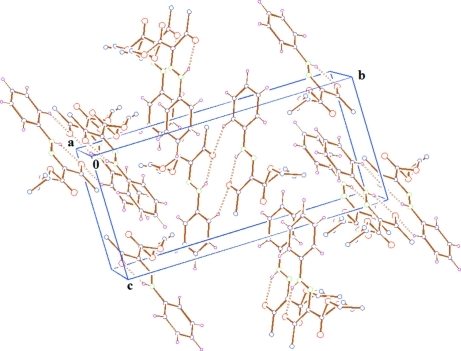
Crystal packing of (I) viewed down the a axis.

## supplementary crystallographic information

### Comment

The unit cell of the title compound, (I), was reported by Wang *et al.* (2005, 2007). In the full structure reported here, the ethoxy group is disordered over two orientations with refined occupancies 0.642 (15):0.358 (15). The planes C9—O3A—C10A (plane A) and C9—O3B—C10B (plane B) of the two disordered ethoxy groups are inclined at an angle of 38.3 (17)°. The bonds C10A—C11A and C10B—C11B are bent in opposite directions with atom C11A and C11B deviating from planes A & B by 1.33 (1)Å and -1.41 (2)Å, respectively. The torsion angles C9—O3A—C10A—C11A and C9—O3B—C10B—C11B are 80.3 (6) and -89.0 (12)°, respectively. An intramolecular N—H···O hydrogen bond contributes to the planarity of the C1···C9, N1, N2, 1-phenyl-2-(propan-2-ylidene)hydrazine segment of the molecule.  In the crystal structure C2—H2···O1 hydrogen bonds link pairs of molecules into centrosymmetric dimers generating R_2_^2^(16) rings (Bernstein *et al.*, 1995).

### Experimental

The title compound was prepared by the coupling of diazonium salt of aniline with ethyl acetoacetate (Fernandes *et al.*, 1975). It was recrystalized from methanol by slow evaporation at room temperature to yield colourless blocks of (I).

### Refinement

High values of isotropic thermal parameters for atoms O3 and C10 and unacceptable bond lengths for O3—C10 and C10—C11 of the ethoxy group indicated possible disorder. A difference elctron density map excluding the atoms O3, C10 and C11 showed that the ethoxy group to be disordered over two sites. The ratio of the occupancy factors of the two disorder components refined to 0.642 (15):0.358 (15). All H-atoms were positioned geometrically and refined using a riding model with d(C-H) = 0.93Å,  U_iso_=1.2U_eq_ (C) for aromatic 0.97Å,  U_iso_ = 1.2U_eq_ (C) for CH_2_, 0.86Å,  U_iso_ = 1.2U_eq_ (N) for NH, and 0.96Å,  U_iso_ = 1.5U_eq_ (C) for CH_3_ atoms.

### Crystal data

**Table:**

C_12_H_14_N_2_O_3_	*F*(000) = 496
*M**_r_* = 234.25	*D*_x_ = 1.275 Mg m^−^^3^
Monoclinic, *P*2_1_/*c*	Cu *K*α radiation, λ = 1.5418 Å
Hall symbol: -P 2ybc	Cell parameters from 25 reflections
*a* = 8.4375 (9) Å	θ = 25.8–35.5°
*b* = 17.551 (2) Å	µ = 0.77 mm^−^^1^
*c* = 8.242 (1) Å	*T* = 293 K
β = 91.24 (1)°	Block, colourless
*V* = 1220.2 (2) Å^3^	0.2 × 0.16 × 0.12 mm
*Z* = 4	

### Data collection

**Table:**

Enraf–Nonius CAD-4 diffractometer	*R*_int_ = 0.022
Radiation source: fine-focus sealed tube	θ_max_ = 69.8°, θ_min_ = 5.0°
graphite	*h* = −10→10
ω–2θ scans	*k* = 0→21
2393 measured reflections	*l* = 0→9
2243 independent reflections	3 standard reflections every 60 min
1715 reflections with *I* > 2σ(*I*)	intensity decay: 0.0%

### Refinement

**Table:**

Refinement on *F*^2^	Secondary atom site location: difference Fourier map
Least-squares matrix: full	Hydrogen site location: inferred from neighbouring sites
*R*[*F*^2^ > 2σ(*F*^2^)] = 0.052	H-atom parameters constrained
*wR*(*F*^2^) = 0.185	*w* = 1/[σ^2^(*F*_o_^2^) + (0.1173*P*)^2^ + 0.0072*P*] where *P* = (*F*_o_^2^ + 2*F*_c_^2^)/3
*S* = 1.07	(Δ/σ)_max_ < 0.001
2243 reflections	Δρ_max_ = 0.19 e Å^−^^3^
186 parameters	Δρ_min_ = −0.17 e Å^−^^3^
0 restraints	Extinction correction: *SHELXL97* (Sheldrick, 2008), Fc^*^=kFc[1+0.001xFc^2^λ^3^/sin(2θ)]^-1/4^
Primary atom site location: structure-invariant direct methods	Extinction coefficient: 0.072 (5)

### Special details

**Table:**

Geometry. All su's are estimated using the full covariance matrix. The cell su's are taken into account individually in the estimation of su's in distances, angles and torsion angles; correlations between su's in cell parameters are only used when they are defined by crystal symmetry.
Refinement. Refinement of *F*^2^ against ALL reflections. The weighted *R*-factor *wR* and goodness of fit *S* are based on *F*^2^, conventional *R*-factors *R* are based on *F*, with *F* set to zero for negative *F*^2^. The threshold expression of *F*^2^ > 2σ(*F*^2^) is used only for calculating *R*-factors(gt) *etc*. and is not relevant to the choice of reflections for refinement. *R*-factors based on *F*^2^ are statistically about twice as large as those based on *F*, and *R*- factors based on ALL data will be even larger.

### Fractional atomic coordinates and isotropic or equivalent isotropic displacement parameters (Å^2^)

**Table:**

	*x*	*y*	*z*	*U*_iso_*/*U*_eq_	Occ. (<1)
O3A	0.5943 (5)	0.3242 (3)	0.4120 (7)	0.0880 (14)	0.642 (15)
C10A	0.7449 (8)	0.2909 (5)	0.3648 (8)	0.104 (2)	0.642 (15)
H10A	0.7582	0.2984	0.2493	0.125*	0.642 (15)
H10B	0.7425	0.2365	0.3851	0.125*	0.642 (15)
C11A	0.8803 (11)	0.3242 (4)	0.4529 (7)	0.117 (2)	0.642 (15)
H11A	0.8555	0.3295	0.5655	0.176*	0.642 (15)
H11B	0.9035	0.3734	0.4084	0.176*	0.642 (15)
H11C	0.9709	0.2916	0.4426	0.176*	0.642 (15)
O3B	0.6366 (10)	0.3601 (9)	0.4633 (10)	0.095 (3)	0.358 (15)
C10B	0.792 (2)	0.3348 (9)	0.416 (2)	0.126 (4)	0.358 (15)
H10C	0.8323	0.3678	0.3320	0.151*	0.358 (15)
H10D	0.8649	0.3363	0.5084	0.151*	0.358 (15)
C11B	0.7762 (19)	0.2558 (11)	0.354 (2)	0.168 (7)	0.358 (15)
H11D	0.7167	0.2560	0.2538	0.252*	0.358 (15)
H11E	0.7222	0.2252	0.4324	0.252*	0.358 (15)
H11F	0.8796	0.2349	0.3371	0.252*	0.358 (15)
O1	0.1739 (2)	0.49615 (9)	0.38454 (16)	0.0898 (5)	
O2	0.6193 (2)	0.41989 (13)	0.2324 (2)	0.1178 (7)	
N1	0.23998 (17)	0.41137 (8)	0.63121 (16)	0.0619 (4)	
H1	0.1711	0.4412	0.5862	0.074*	
N2	0.36686 (18)	0.39265 (8)	0.55505 (17)	0.0621 (4)	
C1	0.2155 (2)	0.38255 (9)	0.78858 (19)	0.0585 (5)	
C2	0.0821 (2)	0.40648 (12)	0.8691 (2)	0.0727 (5)	
H2	0.0112	0.4404	0.8199	0.087*	
C3	0.0557 (3)	0.37944 (14)	1.0233 (3)	0.0833 (6)	
H3	−0.0327	0.3958	1.0790	0.100*	
C4	0.1590 (3)	0.32856 (12)	1.0954 (2)	0.0803 (6)	
H4	0.1396	0.3101	1.1989	0.096*	
C5	0.2912 (3)	0.30493 (11)	1.0145 (2)	0.0742 (6)	
H5	0.3613	0.2707	1.0638	0.089*	
C6	0.3204 (2)	0.33190 (10)	0.8599 (2)	0.0647 (5)	
H6	0.4097	0.3160	0.8051	0.078*	
C7	0.4007 (2)	0.42190 (11)	0.4118 (2)	0.0647 (5)	
C8	0.3017 (2)	0.47900 (11)	0.3269 (2)	0.0700 (5)	
C9	0.5507 (3)	0.39304 (14)	0.3460 (3)	0.0820 (6)	
C12	0.3544 (3)	0.51477 (14)	0.1731 (3)	0.0881 (7)	
H12A	0.2856	0.5565	0.1454	0.132*	
H12B	0.3507	0.4777	0.0875	0.132*	
H12C	0.4610	0.5331	0.1873	0.132*	

### Atomic displacement parameters (Å^2^)

**Table:**

	*U*^11^	*U*^22^	*U*^33^	*U*^12^	*U*^13^	*U*^23^
O3A	0.0801 (18)	0.079 (2)	0.106 (3)	0.0054 (16)	0.0292 (17)	0.0026 (18)
C10A	0.107 (4)	0.093 (4)	0.115 (4)	0.023 (3)	0.040 (3)	−0.008 (3)
C11A	0.087 (4)	0.142 (5)	0.122 (4)	0.029 (3)	0.018 (3)	0.003 (3)
O3B	0.081 (3)	0.111 (7)	0.095 (4)	0.020 (4)	0.020 (3)	0.005 (4)
C10B	0.085 (9)	0.139 (10)	0.154 (10)	0.025 (7)	0.033 (7)	−0.010 (8)
C11B	0.137 (10)	0.129 (12)	0.241 (16)	0.046 (9)	0.066 (9)	−0.030 (10)
O1	0.1019 (11)	0.1033 (11)	0.0645 (8)	0.0229 (8)	0.0097 (7)	0.0206 (7)
O2	0.1127 (14)	0.1545 (18)	0.0879 (12)	0.0112 (11)	0.0391 (10)	0.0288 (11)
N1	0.0694 (9)	0.0669 (9)	0.0495 (8)	0.0048 (6)	0.0022 (6)	0.0060 (6)
N2	0.0684 (9)	0.0645 (8)	0.0535 (8)	−0.0050 (6)	0.0021 (6)	−0.0015 (6)
C1	0.0697 (10)	0.0578 (9)	0.0479 (9)	−0.0059 (7)	−0.0021 (7)	0.0010 (7)
C2	0.0725 (11)	0.0827 (12)	0.0631 (10)	0.0069 (9)	0.0064 (8)	0.0105 (9)
C3	0.0880 (13)	0.0957 (14)	0.0669 (12)	0.0012 (11)	0.0180 (10)	0.0088 (10)
C4	0.1020 (15)	0.0811 (13)	0.0579 (10)	−0.0139 (10)	0.0066 (10)	0.0127 (9)
C5	0.0991 (14)	0.0629 (11)	0.0601 (10)	−0.0009 (9)	−0.0064 (9)	0.0089 (8)
C6	0.0786 (11)	0.0583 (9)	0.0572 (10)	0.0041 (7)	−0.0002 (8)	−0.0005 (7)
C7	0.0736 (11)	0.0695 (10)	0.0511 (9)	−0.0098 (8)	0.0023 (7)	−0.0005 (7)
C8	0.0816 (12)	0.0753 (11)	0.0529 (9)	−0.0100 (9)	−0.0013 (8)	0.0033 (8)
C9	0.0801 (13)	0.1015 (16)	0.0648 (11)	−0.0064 (11)	0.0107 (10)	−0.0009 (10)
C12	0.0926 (15)	0.1040 (16)	0.0677 (12)	−0.0173 (12)	−0.0002 (10)	0.0252 (11)

### Geometric parameters (Å, °)

**Table:**

O3A—C9	1.373 (4)	N1—H1	0.8600
O3A—C10A	1.459 (8)	N2—C7	1.324 (2)
C10A—C11A	1.462 (12)	C1—C2	1.384 (3)
C10A—H10A	0.9700	C1—C6	1.377 (2)
C10A—H10B	0.9700	C2—C3	1.379 (3)
C11A—H11A	0.9600	C2—H2	0.9300
C11A—H11B	0.9600	C3—C4	1.374 (3)
C11A—H11C	0.9600	C3—H3	0.9300
O3B—C9	1.328 (7)	C4—C5	1.376 (3)
O3B—C10B	1.445 (15)	C4—H4	0.9300
C10B—C11B	1.48 (2)	C5—C6	1.387 (3)
C10B—H10C	0.9700	C5—H5	0.9300
C10B—H10D	0.9700	C6—H6	0.9300
C11B—H11D	0.9600	C7—C8	1.472 (3)
C11B—H11E	0.9600	C7—C9	1.477 (3)
C11B—H11F	0.9600	C8—C12	1.491 (3)
O1—C8	1.225 (2)	C12—H12A	0.9600
O2—C9	1.207 (3)	C12—H12B	0.9600
N1—N2	1.295 (2)	C12—H12C	0.9600
N1—C1	1.412 (2)		
			
C9—O3A—C10A	118.3 (4)	C4—C3—C2	120.6 (2)
O3A—C10A—C11A	112.6 (9)	C4—C3—H3	119.7
O3A—C10A—H10A	109.1	C2—C3—H3	119.7
C11A—C10A—H10A	109.1	C5—C4—C3	119.96 (18)
O3A—C10A—H10B	109.1	C5—C4—H4	120.0
C11A—C10A—H10B	109.1	C3—C4—H4	120.0
H10A—C10A—H10B	107.8	C4—C5—C6	120.32 (18)
C9—O3B—C10B	114.9 (9)	C4—C5—H5	119.8
C11B—C10B—O3B	107.8 (17)	C6—C5—H5	119.8
C11B—C10B—H10C	110.1	C1—C6—C5	119.16 (18)
O3B—C10B—H10C	110.1	C1—C6—H6	120.4
C11B—C10B—H10D	110.1	C5—C6—H6	120.4
O3B—C10B—H10D	110.1	N2—C7—C8	123.80 (17)
H10C—C10B—H10D	108.5	N2—C7—C9	113.43 (17)
C10B—C11B—H11D	109.5	C8—C7—C9	122.76 (17)
C10B—C11B—H11E	109.5	O1—C8—C7	118.59 (16)
H11D—C11B—H11E	109.5	O1—C8—C12	120.48 (19)
C10B—C11B—H11F	109.5	C7—C8—C12	120.92 (19)
H11D—C11B—H11F	109.5	O2—C9—O3B	118.1 (4)
H11E—C11B—H11F	109.5	O2—C9—O3A	121.5 (3)
N2—N1—C1	119.56 (14)	O3B—C9—O3A	36.0 (5)
N2—N1—H1	120.2	O2—C9—C7	125.4 (2)
C1—N1—H1	120.2	O3B—C9—C7	109.9 (3)
N1—N2—C7	122.04 (15)	O3A—C9—C7	112.4 (2)
C2—C1—C6	120.85 (16)	C8—C12—H12A	109.5
C2—C1—N1	117.94 (15)	C8—C12—H12B	109.5
C6—C1—N1	121.21 (16)	H12A—C12—H12B	109.5
C1—C2—C3	119.12 (18)	C8—C12—H12C	109.5
C1—C2—H2	120.4	H12A—C12—H12C	109.5
C3—C2—H2	120.4	H12B—C12—H12C	109.5
			
C9—O3A—C10A—C11A	80.3 (6)	C9—C7—C8—O1	175.22 (18)
C9—O3B—C10B—C11B	−89.0 (12)	N2—C7—C8—C12	174.34 (17)
C1—N1—N2—C7	−175.53 (14)	C9—C7—C8—C12	−4.1 (3)
N2—N1—C1—C2	177.16 (15)	C10B—O3B—C9—O2	−22.3 (18)
N2—N1—C1—C6	−3.1 (3)	C10B—O3B—C9—O3A	83.5 (14)
C6—C1—C2—C3	0.6 (3)	C10B—O3B—C9—C7	−175.5 (12)
N1—C1—C2—C3	−179.73 (18)	C10A—O3A—C9—O2	12.4 (9)
C1—C2—C3—C4	−0.9 (4)	C10A—O3A—C9—O3B	−83.2 (8)
C2—C3—C4—C5	0.8 (4)	C10A—O3A—C9—C7	−176.6 (6)
C3—C4—C5—C6	−0.4 (3)	N2—C7—C9—O2	−167.1 (2)
C2—C1—C6—C5	−0.1 (3)	C8—C7—C9—O2	11.5 (3)
N1—C1—C6—C5	−179.79 (16)	N2—C7—C9—O3B	−16.3 (8)
C4—C5—C6—C1	0.0 (3)	C8—C7—C9—O3B	162.3 (8)
N1—N2—C7—C8	1.4 (3)	N2—C7—C9—O3A	22.3 (4)
N1—N2—C7—C9	179.99 (16)	C8—C7—C9—O3A	−159.1 (4)
N2—C7—C8—O1	−6.3 (3)		

### Hydrogen-bond geometry (Å, °)

**Table:**

*D*—H···*A*	*D*—H	H···*A*	*D*···*A*	*D*—H···*A*
N1—H1···O1	0.86	1.92	2.571 (2)	131
C2—H2···O1^i^	0.93	2.53	3.430 (3)	163

Symmetry codes: (i) −*x*, −*y*+1, −*z*+1.
